# Periodically Ordered Wrinkles in Gradient Patterned Polymer Stripes

**DOI:** 10.3390/ma17246035

**Published:** 2024-12-10

**Authors:** Myunghwan Byun

**Affiliations:** Department of Advanced Materials Engineering, Keimyung University, Daegu 42601, Republic of Korea; myunghbyun@kmu.ac.kr; Tel.: +82-53-580-5228

**Keywords:** wrinkles, gradient, controlled evaporative self-assembly

## Abstract

We demonstrated a versatile and robust strategy to create spatially defined periodic wrinkles in gradient striped polymer films through the coupled process of controlled evaporative self-assembly (CESA) and mechanically driven surface wrinkling. The mechanical properties of patterned-gradient polymer thin films were investigated by wrinkling methodology in a fast and simple manner. Understanding of the complex wrinkles can provide insights into the growth mechanism of most biological species that can be explained by competition between bending and stretching energies, e.g., leaves with gradient thickness range from the central area to edges. Furthermore, it also benefits a wide range of micro-to-nanotechnologies that strongly depend on the mechanical stability and performance of thin polymer membranes for semiconductor applications.

## 1. Introduction

Wrinkling in soft materials has received increasing attention due to the ease of using self-organization in such systems to generate well-defined and complex topological features [1]. Spontaneous organization of complex, intriguing, highly ordered structures in simple systems is both arresting and potentially useful in micro-to-nanometer scale technological fields. Wrinkling of thin, high modulus polymer films on a relatively compliant support opens a powerful platform for creating intricate yet well-ordered periodical sinusoidal wave-like patterns that can be controlled and aligned by elastomeric foundation in the surface of the polymer film [2,3,4,5,6,7,8,9,10,11,12,13,14,15,16,17,18]. The periodically riffled structures with characteristic spatial features such as wavelength and amplitude can potentially function as a wide range of practical applications such as diffraction gratings [19,20] and optical sensors [21,22], smart adhesives [23,24,25,26,27], mechanical strain sensors [28,29,30,31,32], microfluidic sorters [33], cell culture surfaces [34], stretchable electronics [35,36,37], thin film metrology [13], etc. In this regard, several pioneering works based upon controlled wrinkling, not as a nuisance but as a ubiquitous platform for creating spatially defined mechanical instabilities, have been reported, including thermally induced wrinkling [2], mechanically driven wrinkling [6,8,9,13], and swelling or drying-mediated wrinkling [5,15]. Most approaches to the formation of wrinkles in selectively patterned areas have been successfully made by the synergy of lithographical techniques for mesoscale patterning of the polymeric thin films and spontaneous wrinkle alignment through well-defined patterns. However, the above-mentioned methods suffer from complex processing steps and high cost. For example, e-beam or deep UV lithography for generating spatially defined capping layers is expensive and sophisticated for its operation and maintenance, although the resulting pattern dimensions are well-controlled. In addition, various topological patterns with different widths and heights require multi-step processing and come with a higher cost. Therefore, fully combining controlled evaporation of confined microfluids as an alternative tool to conventional lithographic technique for generating well-ordered polymer patterns with strain-mismatch induced surface wrinkling may pull off a much simpler and more masterful methodology for structuring of periodic surface wrinkles over patterned areas and provide a new platform for characterizing mechanical properties of patterned polymer films with multi-dimensional scale ranging from micrometers to nanometers. This means that the wrinkles confined in limited areas with a gradient fashion in terms of width and height can be created via a cost-effective and fast method to determine the mechanical properties of various organic or inorganic functional materials including glassy, semicrystalline, conductive or conjugated polymers, block copolymers, graphitic materials, carbon nanotubes, etc., thus establishing a library for characterizing mechanical properties of patterned-gradient polymers.

In the present study, we introduce a simple and robust route to the formation of periodically structured wrinkles in patterned-gradient media enabled by the synergetic process of controlled evaporative self-assembly (CESA) and mechanically driven surface wrinkling. This approach creates a new arena for fundamental studies of how the surface wrinkles can be confined and aligned in patterned areas with gradient fashion in terms of height and width, opening the door to characterization of the material properties of various organic thin film patterns, which would be valuable for designing and implementing flexible and/or stretchable organic electronics.

## 2. Experimental

**Figure 1:**
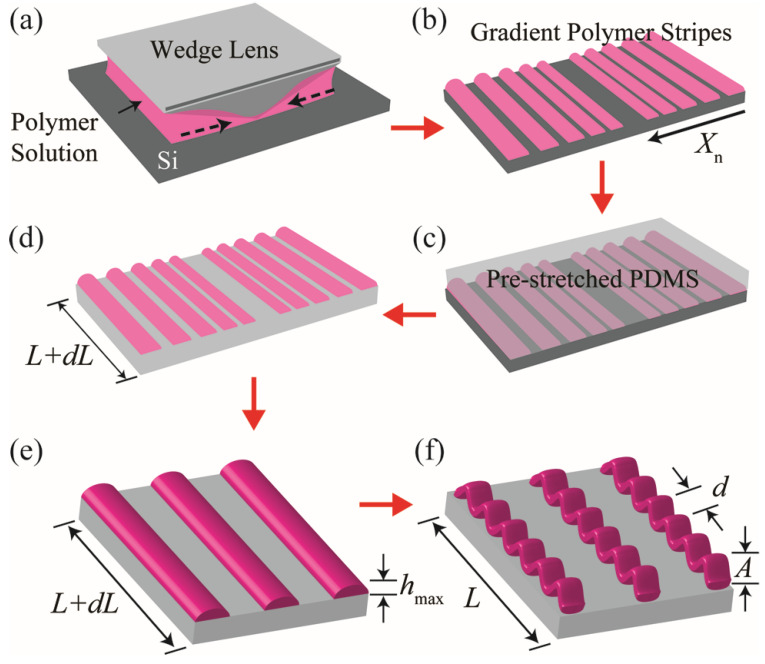
Periodically ordered wrinkles in patterned-gradient polymer stripes. (**a**) Controlled evaporative self-assembly of polymer solution confined in a wedge-on-flat geometry. As schematically illustrated, a drop of PMMA solution is restricted, forming a capillary bridge between the slanted wedge lens and the flat Si substrate. The dotted arrows indicate the shrinkage direction of confined PMMA solution during evaporation. (**b**) Schematic illustration of gradient PMMA stripes. (**c**) Physical contact of a pre-stretched elastomeric PDMS to gradient PMMA stripes. (**d**) Peeling back the PDMS with gradient PMMA stripes transferred from the Si substrate. (**e**,**f**) Schematic illustration of the formation of highly periodic wrinkles in patterned stripes. By releasing the pre-strain, periodically ordered wrinkles are spontaneously formed in the patterned-gradient PMMA stripes.

We developed a versatile and robust approach for the assembly of periodic wrinkles in gradient patterned areas using the process schematically summarized as 1% tensile d in Figure 1. Our approach is in complete contrast to previously reported studies on the wrinkling behaviors of heterogeneously patterned films produced by stamping [38] or lithographic patterning [39], which can barely produce thickness-gradient patterned films.

Wedge-on-Flat Geometry and CESA: A wedge-shaped lens made of aluminum and a flat Si wafer with 2 nm thick native silicon oxide were used as the upper and lower surfaces, respectively, which is called “wedge-on-flat geometry” as shown in Figure S1. The size of the wedge and the wedge height were 1 × 1 cm^2^ (≈the meniscus size) and 1000 µm, respectively. The silicon substrate was vigorously cleaned using a mixture of sulfuric acid and NOCHROMIX, and then clearly rinsed with deionized water and blown dry with nitrogen gas. The wedge-on-flat geometry was placed in a home-made sealed chamber to minimize possible unexpected variables raised from air convection and temperature change during the controlled evaporative self-assembly (CESA) process. Poly(methyl methacrylate) (PMMA, Mn = 534 kg mol^−1^, PDI = 1.57, Sigma Aldrich, St. Louis, MO, USA) was selected to prepare 0.25 mg mL^−1^ PMMA toluene solution. Gradient polymer stripes of PMMA are systematically created by controlled evaporative self-assembly (CESA) based on repetitive pinning/depinning cycles of a three-phase contact line when a drop of polymer solution is forced to evaporate in the wedge-on-flat geometry, as described elsewhere [40,41]. As a result, solvent evaporation is expected to be fastest at the capillary edge. As a volatile solvent becomes depleted, a three-phase contact line at the capillary edge is subject to pinning by spontaneous transportation of nonvolatile polymer molecules from the contact center of the upper wedge and lower flat Si substrate (i.e., “stick”). During this process, the initial contact angle of the meniscus gradually reaches a critical value due to evaporative loss of solvent, at which the depinning force (i.e., capillary force) become larger than the pinning force, causing the contact line to jerk toward the wedge/Si contact center (i.e., “slip”) and become arrested at a new position, thereby leaving behind a straight line pattern [40,41,42,43,44,45,46,47].

Wrinkling Formation: The gradient polymer stripes of PMMA are readily transferred onto a soft, elastomeric polydimethyl siloxane (PDMS) substrate (Figure 1c) with uniaxial tensile pre-strain (dL) (Figure 1d). For preparing the PDMS, a Sylgard 184 (Dow Corning, Midland, MI, USA) silicon base and a curing agent were mixed at a 10:1 ratio by weight. Then the resulting mixture was degassed to remove any tiny air bubbles and poured into the petri dish. The PDMS was baked under a vacuum for curing and to evacuate all entrapped air bubbles. After cooling, the cured PDMS was peeled and cut. The PDMS substrate was directly used without additional surface modification such as plasma or oxygen treatment. When the applied tensile pre-strain is released, the gradient polymer stripes begin to wrinkle periodically with a spatially defined wavelength (d), and amplitude (A), as schematically illustrated in Figure 1f. The wavelength of the wrinkles assembled in the patterned-gradient stripes changes linearly with the maximum thickness (h_max_) of the polymer stripe (d~h_max_).

Characterization of Surface Morphologies: The PMMA stripes formed on the flat Si substrate and the wrinkles were thoroughly examined using an optical microscope (OM; BX51 optical microscope in reflection mode (Olympus, Shinjuku City, Tokyo)) and atomic force microscope (AFM; Dimension 3100 scanning force microscope in tapping mode (Digital Instrument, Santa Barbara, CA, USA)). BS-tap 300 tips (Budget Sensors, Sofia, Bulgaria) with spring constants ranging from 20 to 75 Nm^−1^ were used as scanning probes.

## 3. Results and Discussion

**Figure 2:**
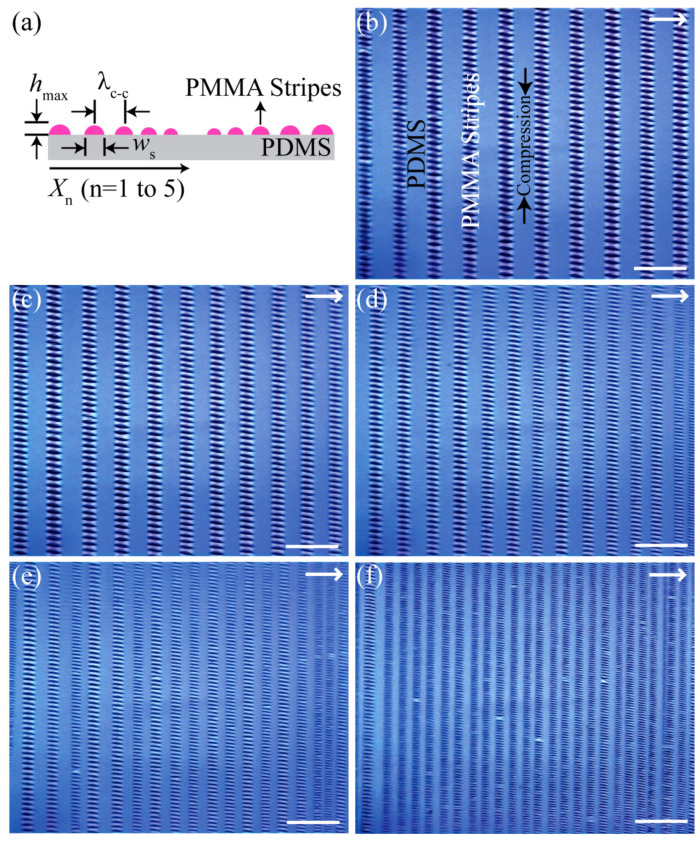
(**a**) Side view of PMMA stripes exhibiting a gradient fashion in the center-to-center distance between the adjacent stripes, λ_c-c_, and the height/width of the stripe, h_max_/w_s_, from outermost to innermost, X_n_ (n = 1 to 5). (**b**–**f**) Optical micrographs obtained at five regions (b = X_1_, c = X_2_, d = X_3_, e = X_4_, and f = X_5_). The arrows indicate the moving direction of the contact line. The scale bars = 100 μm.

**Figure 3:**
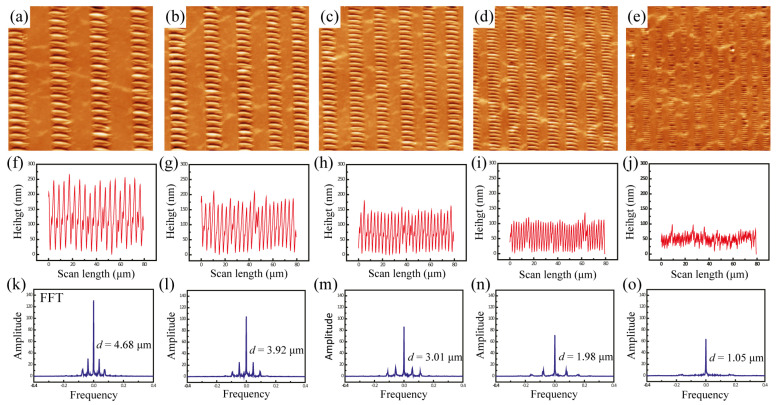
(**a**–**e**) Representative AFM height images of wrinkles over selected five regions (i.e., (**a**) outermost region, X_1_; outermost-intermediate region, X_2_; intermediate region, X_3_, intermediate-innermost region, X_4_; innermost region, X_5_). The scan size = 80 × 80 μm^2^. (**f**–**j**) The corresponding cross-sectional analysis of AFM height images of PMMA wrinkles. (**k**–**o**) Two-dimensional (2D) FFT analysis of the wrinkled topography assuring the improved sensitivity for periodically ordered structures within the patterned-gradient polymer stripes.

Figure 2 obviously visualizes mesoscale wrinkles periodically assembled in the patterned-gradient poly(methyl methacrylate) (PMMA) stripes supported by an elastomeric poly(dimethyl siloxane) (PDMS) foundation. As schematically depicted in Figure 2a, the initial PMMA stripes were in a gradient fashion in terms of thickness (h_max_), width (w_s_), and center-to-center spacing (λ_c-c_), which were measured to be 96 ± 4 nm, 11.4 ± 0.3 µm, and 22.8 ± 0.7 µm at X_1_, 78 ± 4 nm, 9.1 ± 0.1 µm, and 16.0 ± 1.7 µm at X_2_, 62 ± 3 nm, 8.4 ± 0.2 µm, and 13.8 ± 1.1 µm at X_3_, 50 ± 2 nm, 7.1 ± 0.1 µm, and 10.8 ± 0.5 µm at X_4_, and 28 ± 2 nm, 4.3 ± 0.2 µm, and 7.1 ± 0.4 µm at X_5_, respectively. This gradient fashion can be effectively explained by nonlinear volume loss of the solvent resulting from the imbalance between linear pinning force and nonlinear capillary force during the whole evaporation process [46]. At five regions (i.e., X_1_ to X_5_) marked in the OM images (Figure 2b–f)), spatially ordered wrinkles were crafted in the patterned-gradient stripes. The slight tilt of the wrinkles is presumably due to a mismatch between the long axis of the PMMA line pattern and the direction of the applied compressive force. AFM measurements confirmed that periodically aligned wrinkles formed over the entire area, more clearly showing a gradual decrease in the wrinkle wavelength with decreasing strip width (Figure 3a–e). All characteristic wavelengths of the wrinkles were determined both in real space using the distance averaged over multiple waves displayed in atomic force microscopy images (Figure 3f–j) and in reciprocal space using a fast Fourier transform (FFT; Figure 3k–o). The wrinkle wavelengths (real space and FFT) at five regions were measured to be 4.6 ± 0.2 µm (4.68 µm) at X_1_, 3.6 ± 0.2 µm (3.92 µm) at X_2_, 2.8 ± 0.1 µm (3.01 µm) at X_3_, 2.2 ± 0.1 µm (1.98 µm) at X_4_, and 1.1 ± 0.1 µm (1.05 µm) at X_5_. Two-dimensional (2D) FFT analysis of the wrinkled topography assures the improved sensitivity for periodically ordered domains within the gradient polymer stripes. All wrinkle wavelengths were taken at the maximum thickness levels due to gradient divergence of thickness level in a single stripe (i.e., stripes are not uniformly flat, but topologically semi-elliptical), as schematically described in Figure 2a.

**Figure 4:**
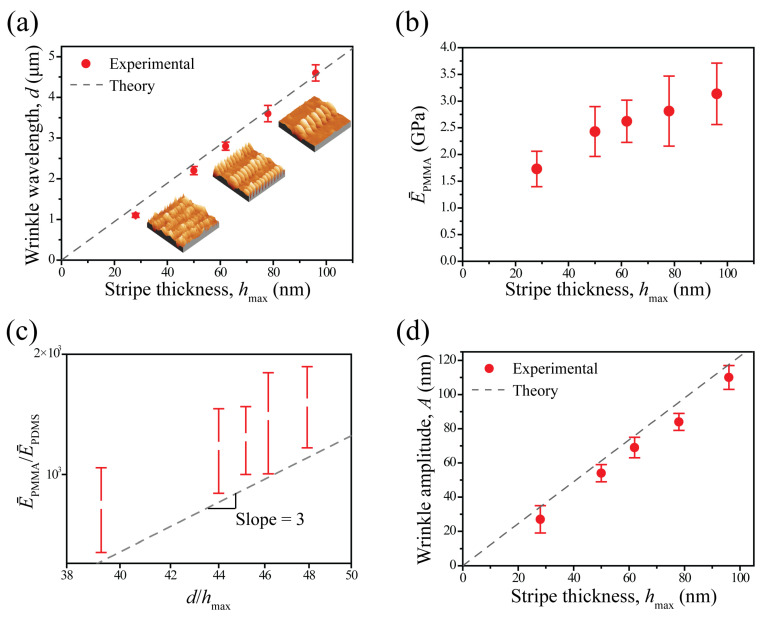
(**a**) All characteristic wavelengths of the wrinkles, d, determined in real space using the distance averaged over multiple waves displayed in AFM images (i.e., Figure 3a–e). The wrinkle wavelength, d, increases with increasing stripe thickness, h_max_. Insets are representative AFM height images obtained at selected areas (i.e., lower = innermost, X_5_; middle = intermediate, X_3_, upper = outermost, X_1_). The dashed line indicates theoretically predicted wavelength, demonstrating good agreement with experimental results. (**b**) Apparent modulus as a function of stripe thickness, h_max_. (**c**) Modulus ratio between top PMMA stripe and bottom PDMS, ${\bar{E}}_{PMMA}/{\bar{E}}_{PDMS}$, as a function of the ratio between wrinkle wavelength, d, and stripe thickness, h_max_. The dashed line indicates theoretical prediction, demonstrating good agreement with experimental results. (**d**) Wrinkle amplitude, A, as a function of stripe thickness, h_max_. The dashed line indicates theoretical prediction, demonstrating good agreement with experimental results. All error bars represent one standard deviation of the data taken as the experimental uncertainty of the measurement.

Generally, surface wrinkles of a thin film supported by a thick, elastomeric foundation can be well-described using an energy balance between the film bending (~Eh^3^) and the in-plain substrate deformation (~Eh^2^). Short wavelengths are suppressed by the sizable bending energy of the thin, stiff film, whereas long wavelengths are suppressed by the large strain energy associated with deformation of the thick, elastomeric foundation [2,3,4,6,13,48]. The characteristic wavelength of the wrinkle, d, is highly expected to be dominant as a function of the initial stripe thickness. A close investigation of the plots displayed in Figure 4a reveals that the wrinkle wavelengths experimentally observed at five regions (solid red circles) show a reasonably close fitting with theoretically predicted values (dotted line) derived from a classic theory $d=2\pi h_{max}{\left({\bar{E}}_{PMMA}/3{\bar{E}}_{PDMS}\right)}^{1/3}$, where h_max_ is the maximum film thickness, $\bar{E}=E/\left(1-\nu^{2}\right)$ is the plain strain modulus (2.34 MPa for PDMS and 2.96 GPa for PMMA), E is Young’s modulus (1.8 MPa for PDMS and 2.6 GPa for PMMA), and ν is Poisson’s ratio (0.48 for PDMS and 0.35 for PMMA). Mechanically induced wrinkling instabilities can be readily used for characterizing the elastic modulus of polymer thin films, as reported in a pioneering work by Stafford et al., which was a study about how the elastic modulus of the polymer thin film with thickness gradient fashion can be measured by applying well-established wrinkling mechanics [13]. In Figure 4b, the plain strain moduli of PMMA stripes (${\bar{E}}_{PMMA}$) at five regions are plotted against their height levels based on the following equation: ${\bar{E}}_{PMMA}=3{\bar{E}}_{PDMS}{\left(d/2\pi h_{max}\right)}^{3}$. The plain modulus of the patterned PMMA stripe gradually decreases with decreasing thickness. This trend is consistent with previous work based on continuous glassy polystyrene (PS) and PMMA thin films on PDMS [14]. In this reference work, they experimentally proved that the modulus of the polymer thin films with thickness ranges from 5 nm to 190 nm, and apparently decreased as the film thickness decreased. In Figure 4c, the plain strain modulus ratio of PMMA and PDMS is plotted against the ratio of wrinkle wavelength and thickness. The relationship between the two quantities is well described by a linear dependence and the best-fit power law is 2.98 ± 0.16, verifying the theoretical prediction that ${\bar{E}}_{PMMA}/{\bar{E}}_{PDMS}\sim {\left(d/h_{max}\right)}^{3}$. A key parameter is the amount of strain required to induce wrinkling in the system. The critical strain can be calculated by the modulus ratio of the substrate and film as follows: $\varepsilon_{c}=1/4{\left(3{\bar{E}}_{s}/{\bar{E}}_{f}\right)}^{2/3}$. It is worth noting that $\varepsilon_{c}$ is not mainly affected by the film thickness. Once strain is externally applied beyond a critical point and thus wrinkles with a specific range of wavelength are formed, the exceeding strain should be accommodated by increasing the wrinkle amplitude (A), whereas the wrinkle wavelength should theoretically remain constant since the wrinkle wavelength is not affected by the amount of applied stress based on a classic theory, not experimentally [48]. In this study, the critical strain for wrinkling formation of the gradient patterned PMMA stripes was calculated to be 0.4% and thus 1% tensile strain was externally applied. In Figure 4d, the wrinkle amplitudes experimentally measured at five regions (solid red circles; 110 ± 7 nm at X_1_, 84 ± 5 nm at X_2_, 69 ± 6 nm at X_3_, 54 ± 5 nm at X_4_, and 27 ± 8 nm at X_5_) show a reasonable fitting with theoretically predicted values (dotted line) based on a classic theory, $A=h_{max}{\left(\varepsilon_{a}/\varepsilon_{c}-1\right)}^{1/2}$, which is very close to the best-fit power law component (1.09 ± 0.05). All characteristic amplitudes of the wrinkles were determined in real space using the half-depth averaged over multiple waves displayed in the AFM images. The amplitudes of the wrinkles formed in gradient patterned stripes decreased as a function of the maximum stripe thickness, as expected from wrinkling mechanics for constant applied strain [2,3,4,6,13,48].

**Figure 5:**
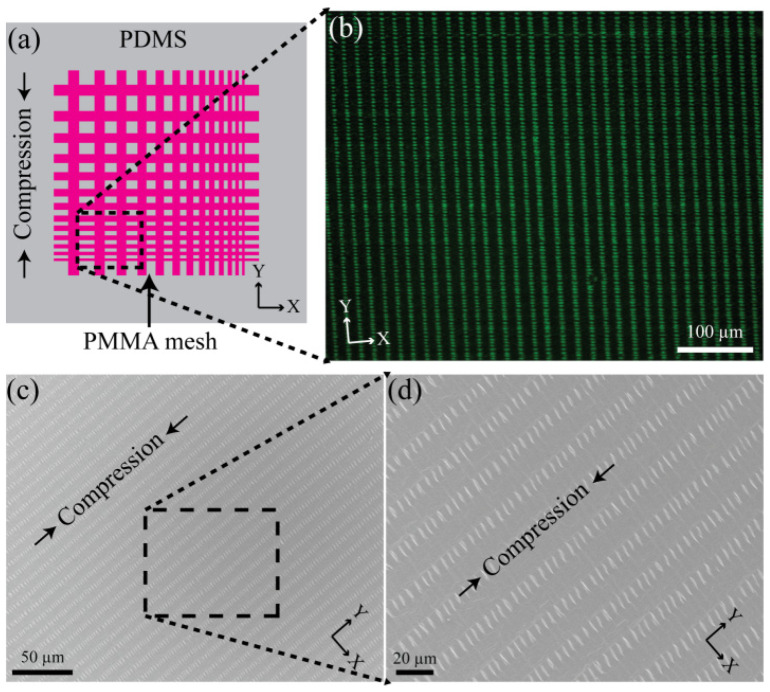
(**a**) Schematic illustration of the formation of PMMA mesh patterns comprising gradient patterned PMMA stripes double layered onto a pre-stretched PDMS. Compressive force was applied parallel to the axis of the 2nd transferred PMMA stripes (i.e., along the Y direction). (**b**) A representative OM image of confined wrinkles taken from the overlaid web pattern of the outermost region (X_1_) in the 1st transferred stripes and the innermost region (X_5_) in the 2nd transferred stripes. (**c**) The corresponding SEM image of (**b**) shows the wrinkles locally confined within the open areas of the 1st transferred PMMA stripes and no wrinkles in intersection areas. (**d**) Zoom-in view of a selected area in (**c**) clearly showing the formation of confined wrinkles.

Well-ordered gradient concentric polymer stripes obtained by controlled evaporative self-assembly (CESA) of a confined microfluid in the curve-on-flat geometry can be readily multi-transferred on to a pre-stretched flexible substrate to generate wrinkles within finite areas. As illustrated in Figure 5, to study confinement of the wrinkles, the first transfer of the CESA PMMA stripes were made and then the second stripes were successively overlaid normal to the axis of the first transferred stripes. Figure 5a shows a schematic cartoon for the formation of PMMA mesh patterns comprised of gradient patterned PMMA stripes double layered onto a pre-stretched PDMS. Compressive force was applied parallel to the axis of the 2nd transferred PMMA stripes, i.e., normal to the 1st transferred PMMA stripes. Figure 5b is a representative optical micrograph of confined wrinkles taken from the overlaid web pattern of the outermost region (X_1_) in the 1st transferred stripes and the innermost region (X_5_) in the 2nd transferred stripes. As clearly shown in a representative SEM image of Figure 5c,d, the wrinkles were locally confined within the open areas of the 1st transferred PMMA stripes, whereas in intersection areas, no wrinkles were formed. Topological features of the wrinkles strongly depend on thickness and lateral size of the open area of the 1st transferred PMMA stripe. In addition, the number of confined wrinkles decreased with decreasing area. Apparently, only one wrinkle was formed. This is a simple and robust approach to hierarchical assembly of confined wrinkles, dispensing with the need for complex and expensive conventional lithographical or external fields for micro-to-nanometer scale patterning of the target films.

## 4. Summary

In summary, we successfully demonstrated a versatile and robust route to the formation of periodically aligned wrinkles through the coupled process of controlled evaporative self-assembly (CESA) as a mesoscale patterning of nonvolatile organic elements and mechano-dynamically driven wrinkling originated from the mismatch between equilibrium strains in a thin, stiff epidermis coupled with a relatively softer, thicker dermis. This process serves as a simple and rapid tool for creating topographically defined areas with a gradient fashion in terms of the height on pre-stretched compliant substrate by transferring patterned-gradient polymer thin films onto a pre-stretched elastomeric foundation. The spatially defined moduli-mismatch regions lead to the controlled alignment of surface wrinkles in patterned-gradient polymer films, which is very sensitive to the geometrical feature size of the areas (i.e., thickness), opening a cost-effective and fast method to determine the mechanical properties of various organic or inorganic functional materials including glassy, semicrystalline, conductive, or conjugated polymers, block copolymers, graphenes or graphites, carbon nanotubes, etc. Spatially defined polymer patterns obtained from controlled evaporation of capillary-held microfluids can be further used as templates for generating patterned-gradient metallic wrinkles. The biggest advantage of the proposed approach is to save cost and time.

## Data Availability

The original contributions presented in this study are included in the article. Further inquiries can be directed to the corresponding author.

## Supplementary Materials

The following supporting information can be downloaded at: https://www.mdpi.com/article/10.3390/ma17246035/s1, Figure S1: A digital image of wedge-on-flat geometry set-up applied in the present study (top). A PMMA toluene solution is trapped between the upper wedge lens and the flat Si wafer. Red colored arrows indicate the direction of the meniscus depression by evaporation. A representative optical microscope image of the PMMA stripes produced via controlled evaporative self-assembly (CESA) process (bottom). Stipe width and spacing between the adjacent stripes decrease from the right to the left sides. The scale bar is 100 μm.
