# Supplementary material for: Periodically Ordered Wrinkles in Gradient Patterned Polymer Stripes
Source: Materials (Basel). 2024 Dec 10;17(24):6035. doi: 10.3390/ma17246035 (PMC11676037; doi:10.3390/ma17246035)
Supplement: Supplementary file 1 [file materials-17-06035-s001.zip › materials-3344769-supplementary.pdf]

Supporting Information

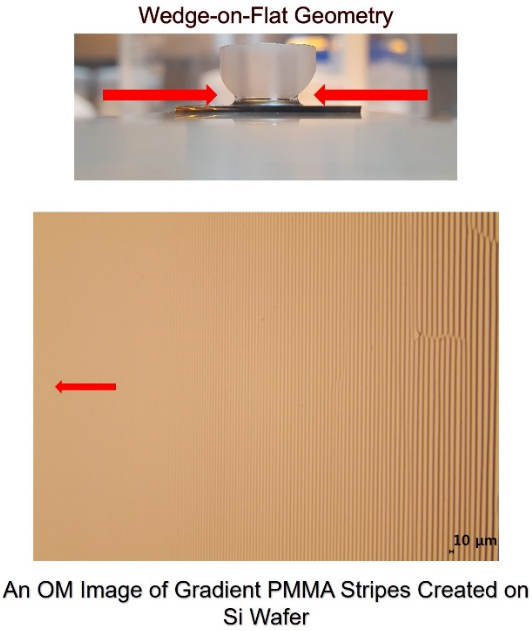

Figure S1. A digital image of wedge-on-flat geometry set-up applied in the present study (top). A PMMA toluene solution is trapped between the upper wedge lens and the flat Si wafer. Red colored arrows indicate the direction of the meniscus depression by evaporation. A representative optical microscope image of the PMMA stripes produced via controlled evaporative self-assembly (CESA) process (bottom). Stipe width and spacing between the adjacent stripes decrease from the right to the left sides. The scale bar is 100 µm.
